# Image-guided breast biopsy and localisation: recommendations for information to women and referring physicians by the European Society of Breast Imaging

**DOI:** 10.1186/s13244-019-0803-x

**Published:** 2020-02-05

**Authors:** Ulrich Bick, Rubina M. Trimboli, Alexandra Athanasiou, Corinne Balleyguier, Pascal A. T. Baltzer, Maria Bernathova, Krisztina Borbély, Boris Brkljacic, Luca A. Carbonaro, Paola Clauser, Enrico Cassano, Catherine Colin, Gul Esen, Andrew Evans, Eva M. Fallenberg, Michael H. Fuchsjaeger, Fiona J. Gilbert, Thomas H. Helbich, Sylvia H. Heywang-Köbrunner, Michel Herranz, Karen Kinkel, Fleur Kilburn-Toppin, Christiane K. Kuhl, Mihai Lesaru, Marc B. I. Lobbes, Ritse M. Mann, Laura Martincich, Pietro Panizza, Federica Pediconi, Ruud M. Pijnappel, Katja Pinker, Simone Schiaffino, Tamar Sella, Isabelle Thomassin-Naggara, Anne Tardivon, Chantal Van Ongeval, Matthew G. Wallis, Sophia Zackrisson, Gabor Forrai, Julia Camps Herrero, Francesco Sardanelli, for the European Society of Breast Imaging (EUSOBI), with language review by Europa Donna–The European Breast Cancer Coalition

**Affiliations:** 1 Clinic of Radiology, Charité Universitätsmedizin Berlin, 10117 Berlin, Germany; 2 PhD Course in Integrative Biomedical Research, Department of Biomedical Science for Health, Università degli Studi di Milano, Via Mangiagalli, 31, 20133 Milan, Italy; 3 Breast Imaging Department, MITERA Hospital, 6, Erithrou Stavrou Str. 151 23 Marousi, Athens, Greece; 4 Department of Radiology, Gustave-Roussy Cancer Campus, 114 Rue Edouard Vaillant, 94800 Villejuif, France; 5 Department of Biomedical Imaging and Image-guided Therapy, Division of Molecular and Gender Imaging, Medical University of Vienna, Waehringer Guertel 18-20, 1090 Wien, Austria; 6 MH EK Honvédkórház, Róbert károly krt. 44, Budapest, 1134 Hungary; 7 Department of Diagnostic and Interventional Radiology, University Hospital Dubrava, University of Zagreb School of Medicine, Zagreb, Croatia; 8 Unit of Radiology, IRCCS Policlinico San Donato, San Donato Milanese, Milan, Italy; 9 Breast Imaging Division, European Institute of Oncology, Milan, Italy; 10 Radiology Unit, Hospices Civils de Lyon, Centre Hospitalo-Universitaire Femme Mère Enfant, 59 Boulevard Pinel, 69 677 Bron Cedex, France; 11 School of Medicine, Department of Radiology, Acıbadem Mehmet Ali Aydınlar University, Istanbul, Turkey; 12 Dundee Cancer Centre, Clinical Research Centre, Ninewells Hospital and Medical School, Tom McDonald Avenue, Dundee, UK; 13 Diagnostic and Interventional Breast Imaging, Department of Radiology, University Hospital, LMU Munich, Marchioninistr. 15, 81377 Munich, Germany; 14 Division of General Radiology, Department of Radiology, Medical University Graz, Auenbruggerplatz 9, 8036 Graz, Austria; 15 Department of Radiology, University of Cambridge, Cambridge Biomedical Campus, Hills road, Cambridge, CB2 0QQ UK; 16 Referenzzentrum Mammographie München and FFB gGmbH München, Sonnenstraße 29, 80331 Munich, Germany; 17 CyclotronUnit, GALARIA-SERGAS, Nuclear Medicine Department and Molecular ImagingGroup, Instituto de Investigación Sanitaria (IDIS), Santiago de Compostela, Spain; 18 Institut de Radiologie, Clinique des Grangettes, Chemin des Grangettes 7, 1224 Chêne-Bougeries, Genève, Switzerland; 19 University Hospital of Aachen, Rheinisch-Westfälische Technische Hochschule, Pauwelsstraße 30, 52074 Aachen, Germany; 20 Radiology and Imaging Laboratory, Fundeni Institute, Bucharest, Romania; 21 Department of Radiology, Zuyderland Medical Center, Dr. H. van der Hoffplein 1, PO Box 5500, 6130 MB Sittard-Geleen, The Netherlands; 22 Department of Radiology, Radboud University Nijmegen Medical Centre, Geert Grooteplein Zuid 10, 6525 GA Nijmegen, The Netherlands; 23 Unit of Radiodiagnostics ASL AT, Via Conte Verde 125, 14100 Asti, Italy; 24 Breast Imaging Unit, Scientific Institute (IRCCS) Ospedale San Raffaele, Via Olgettina, 60, 20132 Milan, Italy; 25 Department of Radiological, Oncological and Pathological Sciences, Sapienza University of Rome, Viale Regina Elena, 324, 00161 Rome, Italy; 26 Department of Imaging, University Medical Centre Utrecht, Utrecht University, Heidelberglaan 100, 3584 CX Utrecht, The Netherlands; 27 Department of Radiology, Breast Imaging Service, Memorial Sloan Kettering Cancer Center, 300 E 66th Street, New York, NY 10065 USA; 28 Department of Diagnostic Imaging, Hadassah Hebrew University Medical Center, Jerusalem, Israel; 29 Department of Radiology, Sorbonne Université, APHP, Hôpital Tenon, 4, rue de la Chine, 75020 Paris, France; 30 Department of Radiology, Institut Curie, Paris, France; 31 Department of Radiology, University Hospitals Leuven, Herestraat 49, 3000 Leuven, Belgium; 32 Cambridge Breast Unit and NIHR Biomedical Research Unit, Box 97, Cambridge University Hospitals NHS Foundation Trust, Cambridge Biomedical Campus, Hills Road, Cambridge, CB2 0QQ UK; 33 Diagnostic Radiology, Department of Translational Medicine, Faculty of Medicine, Lund University, Skåne University Hospital Malmö, SE-205 02 Malmö, Sweden; 34 Department of Radiology, Duna Medical Center, Budapest, Hungary; 35 Jefa Corporativa del Área de la Mama, Ribera Salud Grupo, Valencia, Spain; 36 Department of Biomedical Sciences for Health, Università degli Studi di Milano, Via Morandi 30, 20097 San Donato Milanese, Milan, Italy

**Keywords:** Breast, Breast lesion localisation, Core needle biopsy, Fine-needle sampling, Vacuum-assisted biopsy

## Abstract

We summarise here the information to be provided to women and referring physicians about percutaneous breast biopsy and lesion localisation under imaging guidance. After explaining why a preoperative diagnosis with a percutaneous biopsy is preferred to surgical biopsy, we illustrate the criteria used by radiologists for choosing the most appropriate combination of device type for sampling and imaging technique for guidance. Then, we describe the commonly used devices, from *fine*-*needle sampling* to tissue biopsy with larger needles, namely *core needle biopsy* and *vacuum*-*assisted biopsy*, and how *mammography*, *digital breast tomosynthesis*, *ultrasound*, or *magnetic resonance imaging* work for targeting the lesion for sampling or localisation. The differences among the techniques available for localisation (*carbon marking*, *metallic wire*, *radiotracer injection*, *radioactive seed*, *and magnetic seed localisation*) are illustrated. Type and rate of possible complications are described and the issue of concomitant antiplatelet or anticoagulant therapy is also addressed. The importance of *pathological*-*radiological correlation* is highlighted: when evaluating the results of any needle sampling, the radiologist must check the concordance between the cytology/pathology report of the sample and the radiological appearance of the biopsied lesion. We recommend that special attention is paid to a proper and tactful approach when communicating to the woman the need for tissue sampling as well as the possibility of cancer diagnosis, repeat tissue sampling, and or even surgery when tissue sampling shows a lesion with uncertain malignant potential (also referred to as “high-risk” or B3 lesions). Finally, seven frequently asked questions are answered.

## Key points

- Image-guided needle biopsy is a safe and accurate non-surgical method to diagnose suspicious abnormal findings at breast imaging, pivotal for adequate decision-making, including treatment planning.
- Complete and adequate information must be given to the woman before image-guided breast interventions and informed consent should be obtained from the woman before the procedure.
- The combination of device for sampling and image modality for guidance is chosen by the radiologist for each individual case.
- Pathological-radiological correlation, *i.e.,* the check of concordance between cytology/pathology report of the sample and radiological appearance of the lesion, must be performed.
- Image-guided preoperative localisation is mandatory for guiding surgery of nonpalpable lesions or surgically relevant extension of palpable lesions.

## Introduction

Percutaneous *image*-*guided needle biopsy* is essential in the management of suspicious breast lesions detected by screening or during the assessment of clinical abnormalities. It is a safe and cost-effective procedure allowing for an accurate diagnosis, pivotal for adequate decision-making, including, when indicated, treatment planning. Percutaneous image-guided breast biopsies have almost entirely replaced diagnostic surgical excisions that were associated with longer hospital stay, higher cost, and possible complications. In 2010, the European Society of Breast Cancer Specialists, EUSOMA, suggested that 90% of all the women with breast cancer (invasive or ductal carcinoma in situ [DCIS]) should have a preoperative diagnosis by means of percutaneous biopsy [1].

Moreover, the increasing rate of nonpalpable breast lesions detected in screening programmes as well as the general goal of reducing the extent of surgical treatment have increased the need for *localisation before surgery*. Localisation can also be performed when neoadjuvant therapy is under consideration in order to mark the lesion site for re-evaluation and treatment planning. It is routinely performed using same image guidance techniques used for biopsy and allows for conservative surgical excision of a limited amount of tissue, yielding together an effective treatment and good aesthetic results [2, 3].

Different modalities are available for image-guided breast biopsy and localisation procedures, each of them with their own strengths and weaknesses [4–6]. The most appropriate method is chosen by radiologists for each individual case. Breast radiologists covering the full spectrum of breast imaging and percutaneous tissue sampling techniques (including the use of markers) and presurgical localisation methods are the most suitable professionals for choosing the optimal technique. When localising methods imply the use of radiotracers or radioactive seeds, radiopharmacy and radio safety training (or cooperation with nuclear medicine/radiotherapy departments) is needed.

This article is the fifth of a series of recommendations for women’s information, all issued by the European Society of Breast Imaging, EUSOBI, the first [7] and the third [8] focusing on mammography, the second on breast magnetic resonance imaging (MRI) [9], and the fourth on breast ultrasound (US) [10]. The current article represents also an update of a previous EUSOBI guideline regarding diagnostic interventional breast procedures, published in 2007 [11]. It is in particular addressed to patients for whom an image-guided breast biopsy or localisation is, or may be, under consideration and to physicians dealing with these patients. In particular, eight special information notes (from A to H) and seven frequently asked questions (FAQs) are formulated for direct communication with women. Considering the differences across European countries in terms of available technology, national guidelines, clinical practices, health care systems, and insurance coverage, the applications of these recommendations can vary under local conditions.

A search on the PubMed/Medline has been performed for papers published from January 2009 to March 2019, using the terms “breast” AND “biopsy” OR “fine needle” OR “localization” OR “marker” OR “interventional”. Articles with an informative content most suitable for the purpose of these recommendations were selected as references with special regard to predetermined issues: safety/quality, protocols and techniques, test performance (sensitivity and specificity), and clinical indications. Other articles were included when found to be important among the references of the retrieved articles or when suggested by one or more authors. The entire text underwent a double evaluation by the authors, each of them contributing with relevant intellectual content. However, as many different topics are considered, single authors generally agreeing on these recommendations may have different opinions on individual statements.

This article summarises the information to be provided to women and referring physicians about percutaneous breast biopsy and presurgical localisation under the guidance of mammography/tomosynthesis, US, and MRI.

## Why is preoperative diagnosis through a percutaneous biopsy preferred to surgical biopsy?

Currently, even taking into account recent advances in breast imaging, tissue sampling represents the most accurate method for confirmation or exclusion of malignancy [4]. In fact, there are a variety of benign abnormalities which can mimic malignancy on all breast imaging modalities, *i.e.,* mammography and other x-ray techniques (including tomosynthesis and contrast-enhanced mammography), US, and MRI. The reason to perform a percutaneous biopsy is to prevent unnecessary surgery, associated morbity and costs for equivocal findings on imaging with final non-malignant histopathology. In addition, tailored treatment strategies are currently available including chemotherapy and hormonal therapy before or after surgery (so-called neoadjuvant therapy or adjuvant therapy, respectively), surgical options from lumpectomy to mastectomy with immediate reconstruction, whole or partial breast radiation therapy. The choice among all these options is influenced not only by imaging findings (especially in relation to disease extent) but also by the diagnosis based on percutaneous tissue sampling, in particular when the analysis includes not only basic morphological characteristics but also the molecular pattern of the tumour [11]. Moreover, needle sampling of axillary lymph node, when indicated, adds information for treatment planning [12–14].

We will describe here the details of the different technical options for breast tissue sampling—fine-needle sampling (FNS), core needle biopsy (CNB), and vacuum-assisted biopsy (VAB)—and the imaging modalities for guidance.

We highlight the results of the latest systematic review of the literature and meta-analysis, published in 2014 by the United States Agency for Healthcare Research and Quality [15]. Based on 160 studies using CNB and VAB techniques, the authors found that both US-guided and mammography-guided biopsies had average sensitivities over 97% and specificities ranging from 92 to 99% while non-imaging-guided free-hand biopsy methods had an average sensitivity of 91% and specificity of 98%. Considering that free-hand non-imaging-guided biopsies are performed only on large palpable lesions, image-guided biopsies had better results for the more demanding task of sampling nonpalpable, smaller lesions. For this reason, *free*-*hand breast biopsy cannot be recommended whenever image*-*guided biopsy is available*. CNB and VAB devices (under the same imaging guidance) had similar performances; CNB was found to be associated with a lower risk of adverse events and complications than open surgical biopsy, which were sparsely reported (*e.g.,* 2–10% haematomas, 4% repeat biopsy, 4–6% infections, 2% abscesses). The incidence of adverse events with CNB was found to be 1–1.5% and that of severe complications less than 1% for all needle sampling techniques; VAB appeared to be associated with increased bleeding and haematoma formation; biopsies performed with patients seated upright appeared to be associated with increased risk of vasovagal reactions; CNB obviated the need for surgery procedures in about 75% of women. The authors concluded that the *evidence suggests that US*-*guided and mammography*-*guided biopsies have sensitivity and specificity close to that of surgical biopsy with fewer adverse events and that non*-*imaging*-*guided free-hand procedures have lower sensitivity than image*-*guided methods*. This large literature review clearly explained the reasons for recommending image-guided biopsy instead of surgical biopsy as a general rule of good practice. However, with regard to VAB versus CNB, lesion size and type must be considered and this may have resulted in an underestimation of the potential advantages of VAB over CNB, particularly when using mammography, tomosynthesis, or MRI guidance.

It is important to note that percutaneous needle biopsy may not provide a definitive diagnosis when the histopathological report describes the presence of a *lesion with uncertain malignant potential* (also called *high*-*risk* or *B3 lesion*). This occurs in 3 to 9% of cases, with a range of rates turning out to be malignant (10–33% or also higher rates) [10, 16–25]. These lesions include atypical ductal hyperplasia, benign phyllodes tumours, flat epithelial atypia, classical lobular neoplasia, papillary lesions, radial scars, and other rare entities. Each of them, when surgically removed, shows a variable upgrade rate to invasive cancer or DCIS [26]. Although a small but significant increase of imaging surveillance has been described for classical lobular neoplasia, flat epithelial atypia, and papillary lesions diagnosed on VAB instead of surgical removal, from 24 to 35% of high-risk/B3 lesions (in particular, atypical ductal hyperplasia and phyllodes tumours), are recommended for surgery [27]. In addition, breast specialists should take into account that women diagnosed with high-risk/B3 lesions have a long-term moderately increased risk of breast cancer [28].

Women should be informed that the radiologist may propose a repeat needle sampling or surgical intervention also in the case of biopsy resulting into normal breast tissues or benign abnormalities. After a negative breast tissue needle sampling, imaging follow-up is usually planned, with imaging modalities and time interval to be defined for each individual case.

***Note A.*** *Percutaneous image-guided biopsy has replaced surgical biopsy allowing a minimally invasive safe, accurate, and cost-effective diagnosis of breast lesions, necessary for the definition of treatment planning. In the case of a biopsy resulting in a pathological diagnosis of a high-risk/B3 lesion, discuss with your radiologist and the breast care team the best option for you (either repeat biopsy, surgical removal, or imaging surveillance). After a negative image-guided tissue sampling, imaging follow-up is usually planned, with imaging modalities and time interval to be defined for each individual case.*

## Options for breast tissue sampling: from thin to thick needles

Different percutaneous image-guided techniques are available to diagnose palpable and nonpalpable breast lesions. In the last decades, they have improved patient management, avoiding unnecessary surgical biopsy for benign lesions [29]. Nowadays, FNS, CNB, and VAB coexist, the first providing material for studying cells (cytological examination), the last two providing material for studying tissues (histopathological examination). All these techniques can be theoretically guided by mammography, tomosynthesis, US, or MRI. However, FNS and CNB should be used under US guidance, VAB under mammography/tomosynthesis or MRI guidance. These prevalent combinations are due to both technical considerations, including visibility at each technique and lesion types detected. Breast size, lesion location and size as well as local availability of instrumentation and expertise are taken into account. Table 1 shows the indications for the combinations between imaging guidance and sampling type.

**Table 1:** Current options for breast tissue sampling: combinations of needle types and imaging guidance

Image guidance/sampling type	Fine needle	Core needle	Vacuum-assisted
Ultrasound	Conditionally indicated^a^	Indicated	Indicated
Mammography/tomosynthesis	Not indicated	Not indicated	Indicated
MRI	Not indicated	Not indicated	Indicated

^a^Fine-needle sampling has specific limitations; it is reliably used by centres having specific local experience (see text)

*Whenever the lesion is well identified on US*, *this technique is preferred due to the easy approach and the short duration of the procedure* (*implying a more comfortable woman*’*s experience*) *as well as a lower cost*. If a lesion is not clearly identifiable on US, mammography, tomosynthesis or MRI is used for guidance, typically the former in the case of suspicious calcifications and architectural distortions and the latter in the case of lesions only visible on MRI [30].

Needles of different size and length are adopted for percutaneous image-guided biopsies. The diameter is described by gauge numbers. *Differently from an intuitive reasoning*, *smaller gauge numbers indicate larger needle diameters*. Commonly applied needles have a diameter ranging from 0.4 (27 gauge) to almost 4.6 mm (7 gauge).

### Fine-needle sampling

As mentioned before, FNS is performed almost only under US guidance. Local anaesthesia can be performed but it is not ubiquitous practice. A fine needle with diameter variable from 27 to 18 gauge (same or similar to those used for intramuscular injections) is inserted very close to the US probe and, once the needle is seen inside the target, a manual multidirectional sampling is performed, through aspiration using a 10-20-mL syringe or a vacuum aspiration system (*fine*-*needle aspiration*) or simply by manual movement of the needle inside the lesion (*fine*-*needle capillary sampling*) for about 10–20 sec. The extracted material is then spread onto slides and placed in formalin for cytological analysis. The procedure is easy, safe, and fast to perform and the associated cost is very low. When the cytopathologist is onsite during the sampling, results may be available very soon after the procedure [31]. The success of the technique is highly dependent on the skills of the physician performing the sampling and of the cytopathologist interpreting the sample as well on their interplay [32]. A meta-analysis published in 2008 [33] reported on 25 studies describing FNS cytology analyses performed from 1984 to 2007 on palpable breast masses. The pooled sensitivity was 93% (range 78–100%) and the pooled specificity 98% (range 76–100%). A significant increase in diagnostic performance was shown during the years and attributed to technologic improvements. However, we should note that the report considered only FNS performed on palpable, *i.e.,* relatively large, masses.

It is not surprising that higher rates of inadequate or false-negative results and lower accuracy rates have been reported for FNS compared to CNB. In fact, relying only on cells, FNS cytology cannot always reliably distinguish between benign tissues, high-risk/B3 lesions, DCIS, and invasive malignant changes. In many centres, the information about tumour biomarker status (especially required when a neoadjuvant treatment is under consideration) cannot be obtained from FNS [11]. For these reasons, FNS has increasingly been replaced by CNB or VAB in the diagnosis of breast lesions [34, 35]. As already said, FNS cytology reliability must be considered strongly depending on local factors, *i.e.,* on the experience of operators as well as the presence of a cytopathologist in the room during the procedure. When its performance is high, it can be used as the first fast approach, using CNB or VAB as second step, when needed.

US-guided fine-needle aspiration is widely accepted for draining *complicated cysts* (*e.g.,* cysts with internal debris), seromas or haematomas, for therapeutic purposes for pain relief from swelling cysts, or in the case of *therapy of lactational and non*-*lactational breast abscesses*, as an effective alternative to surgery [36–39]. In particular, lactational abscesses can be managed by US-guided percutaneous treatment, avoiding surgery even for abscesses greater than 5 cm and allowing continued breastfeeding [38]. In the case of *complex cysts* (thick-walled cystic lesions with or without thick internal septations, intracystic solid masses, and mixed cystic/solid lesions), CNB is preferred [36].

### Core needle biopsy

Ultrasound guidance is the most commonly used approach for CNB. After local anaesthesia is administered through subcutaneous injections of drugs similar to those used by dentists (*e.g.,* lidocaine), a needle with a size usually varying from 16 to 12 gauge (most commonly 14 gauge), is inserted by the radiologist, often through a small skin incision. Once the needle is confirmed to be on target, a tissue sample (*core*) is obtained with a needle of variable length (from about 10 to over 20 mm), depending on the used device, and immediately fixed in small formalin containing jars. Since a lesion may be pushed ahead while shooting, the longer samples obtained from longer acquisition chambers are usually preferred. Biopsy devices utilise a spring-loaded needle (or “gun”) that are semiautomatically or automatically fired into the lesion. *This fire is accompanied by a noise and the patient should be informed of this to avoid movements during the biopsy*. A variable number of cores (usually 3–5) are obtained through subsequent samples and needle extractions. Images are acquired to document the correct needle positioning [40–42]. CNB yields material that can be histologically evaluated; it allows a high rate of adequate sampling and a low false-negative rate; in addition, it allows obtaining biological markers necessary for treatment planning. In the case of US-guided 14-gauge CNB, the false-negative rate has been reported to range from 1.2 to 3.3% (mean 2%) [43].

However, since only a portion is sampled from heterogeneous tissue, the risk of *pathological underestimation* remains. This term means that the needle biopsy may provide a result different from that obtained after surgical removal, typically high-risk/B3 lesion instead of DCIS or invasive cancer, or DCIS instead of invasive cancer.

In 2% of cases [43] CNB may provide a false-negative diagnosis, although this event is less frequent than with FNS. Reasons for false negatives can be errors in lesion targeting (bleeding or lesion movement when the needle is fired), histological malignant/benign heterogeneity of lesion (the samples only contain benign tissues, even though the needle was correctly placed). The latter may occur with lesions with heterogeneous texture or in diffusely growing malignancies, where only single cells or nests of cells are contained in otherwise benign appearing or fibrotic tissue (*e.g.,* DCIS, lobular invasive cancers, diffusely growing invasive ductal cancers).

*The most important step to avoid false*-*negative results after percutaneous breast biopsy is systematic radiologic-pathologic correlation of all benign and high*-*risk/B3 histopathological results by the multidisciplinary team including the pathologist and the radiologist*.

Depending on the underlying histological abnormality, from 10 to 50% of lesions characterised as high-risk/B3 (*i.e.,* lesions with uncertain malignant potential) biopsied using CNB will eventually turn out to be malignant [24], and around 25% of tumours diagnosed as DCIS using CNB will have an invasive component on final surgery [44].

### Vacuum-assisted biopsy

This method uses needles with a size from 12 to 7 gauge, the latter being the largest size commercially available for a percutaneous breast biopsy. After local anaesthesia, through a small incision in the skin, a special needle connected to a vacuum-generating device is inserted into the breast and number of tissue samples are taken. *Multiple samples can be taken sequentially without removing the needle*, *which is different to CNB*. In addition, the vacuum attracts the tissue towards the needle and a rotating device cuts the samples. This approach allows for rapid removal of much larger amounts of tissue in comparison to CNB, *i.e.,* 1 g or 1 cm^3^ of tissue per procedure or more [45, 46], thus reducing (but not nulling) the risk of false-negative results or pathological underestimation [47]. Since blood is suctioned from the biopsy site, the risk of lesion displacement by bleeding is reduced.

The VAB can be performed under mammography, tomosynthesis, US, or MRI guidance. The use of VAB is crucial for findings such as microcalcifications or architectural tissue distortions on mammography or tomosynthesis as well as for suspicious contrast-enhancing lesions on MRI that cannot be found with other methods [48]. With aspiration and rinsing of the cavity, VAB has been established as an appropriate and effective technique for US-guided percutaneous drainage of breast abscesses [38, 39].

When VAB is performed under US guidance, the patient is positioned in the same way as diagnostic examinations, *i.e.,* in a supine position; for MRI-guided VAB, the patient is positioned in prone position. When mammography/tomosynthesis is used for guiding the procedure, both dedicated horizontal tables (with the patient in prone position) or upright systems (with the patient seated or laying on her side on a special table or chair) are available, according to experience and resources of the medical centre, both of them being suitable. *Dedicated prone tables are more expensive than the upright alternatives but are less frequently associated with vasovagal reactions*, *partly because interventions occurring below the table cannot be seen by the patient*.

For mammography- or MRI-guided procedures, once the lesion has been localised on images, a computer determines the spatial coordinates that define the lesion position. After local anaesthesia is administered, the needle is positioned in place. If an US-guided VAB is used, real-time scanning is used to identify the lesion and to reach the target; in the case of mammography or MRI guidance, images are acquired to document the needle position [48]. Multiple (up to 12 or more) tissue cores through a 9-gauge or 12-gauge needle are obtained, or a comparable volume with other needle sizes [47]. A relatively large amount of tissue is required for an accurate histopathological analysis. Small lesions (*e.g.,* less than 1 cm in size) may be completely removed.

### Other breast biopsy systems

In the last decade, percutaneous image-guided systems aiming to completely remove small breast lesions have been developed [49–51], for instance using radiofrequency to excise intact lesions in a basket preserving the tissue structure [52–55]. These devices have the potential to provide highly accurate tissue diagnosis and offer an alternative option for percutaneous lesion removal. However, they are currently mainly used in the context of research studies.

### Post-biopsy marker deployment

A marker, typically a metallic clip visible on mammograms (but also markers visible on US and/or MRI), can be placed in the biopsy site at the end of sampling with CNB or VAB to allow for subsequent checking of concordance between the pathological results and imaging appearance, as well as for preoperative localisation. This is especially important for small lesions that can be completely removed with VAB and are no longer visible after biopsy [56] and also for completely drained cystic lesions. Positioning a marker is also necessary to mark lesions in patients who are candidates for neoadjuvant therapy. In the case of important tumour regression, when lesions may no longer be visible on imaging, the clip indicates the tumour location and can be targeted to excise the tumour bed. A marker is always placed after an MRI-guided biopsy, even if the lesion remains visible after biopsy. This confirms the correct location of the sampling but also allows performance of any subsequent intervention by means of the easier mammographic or US guidance.

For biopsies with marker placement, a post-biopsy mammogram is commonly performed, either immediately following the procedure or later, *e.g.,* at the time when the biopsy results are discussed. This mammogram is useful for confirming lesion targeting (correct or displaced location of the marker in the biopsy site) and assessing reduction or absence of lesion findings (*e.g.,* calcifications) after biopsy. In addition, it provides a comparison for future follow-up exams.

***Note B.*** *When a breast abnormality identified on imaging requires a tissue diagnosis, different biopsy options exist to obtain adequate samples. Among FNS, CNB, and VAB, the radiologist will opt for the best method allowing for an accurate diagnosis, depending on lesion appearance, patient characteristics, and local availability of devices. When needed, a metallic marker at the biopsy site is placed. Don’t worry: these markers are not a contraindication for any MRI examination and do not alarm at the airport!*

## Choosing the optimal imaging guidance

Image guidance outperforms non-imaging-guided free-hand approaches for breast lesion sampling. The choice among mammography/tomosynthesis, US, or MRI methods is made by the radiologist according to several factors, the most important being the visibility of the lesion to be targeted.

### US guidance

**Fig. 1:**
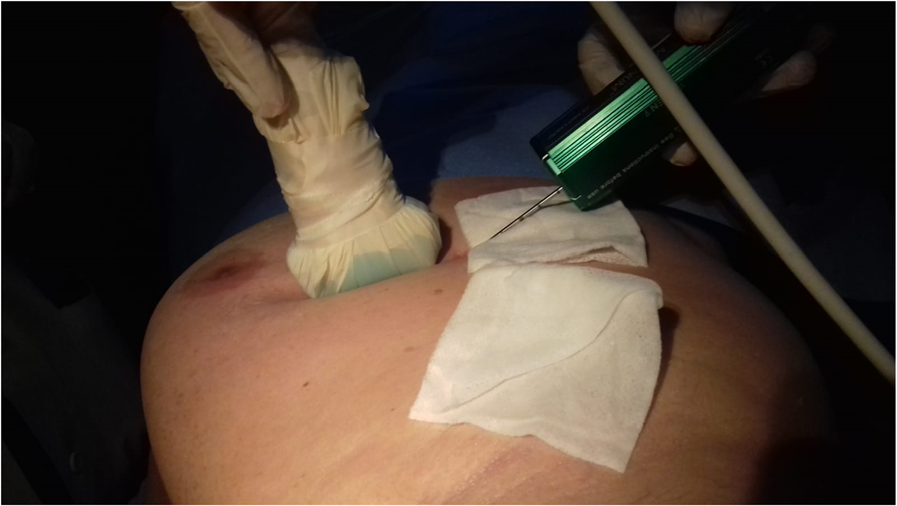
Ultrasound-guided core needle biopsy. The patient is in supine position. After local anaesthesia, the ultrasound probe (on the left) guides the needle to the lesion

If a lesion is visible on US, the best choice is to perform the biopsy under its guidance [57, 58]. US is readily available, does not expose the patient to ionising radiation, and allows for real-time checking of needle placement. US-guided biopsy can also be performed as a bedside procedure in bed-bound patients and there are virtually no contraindications or anatomical/technical restrictions for biopsy access to breast lesions. Typically, US-guided biopsy is performed as CNB, allowing for a safe, fast, effective, and cheap procedure [42, 57], but US is also suited to guide FNS or VAB, taking into account that each system can have specific characteristics [58]. During US-guided biopsy, the patient is usually positioned supine or in the supine oblique position, similar to the US examination (depending on the location of the lesion) (Fig. 1). The duration of a US-guided procedure is about 5 to 15 min, mainly depending on the type of needle used, number of samplings, lesion site, and radiologist’s experience.

### Mammographic guidance

**Fig. 2:**
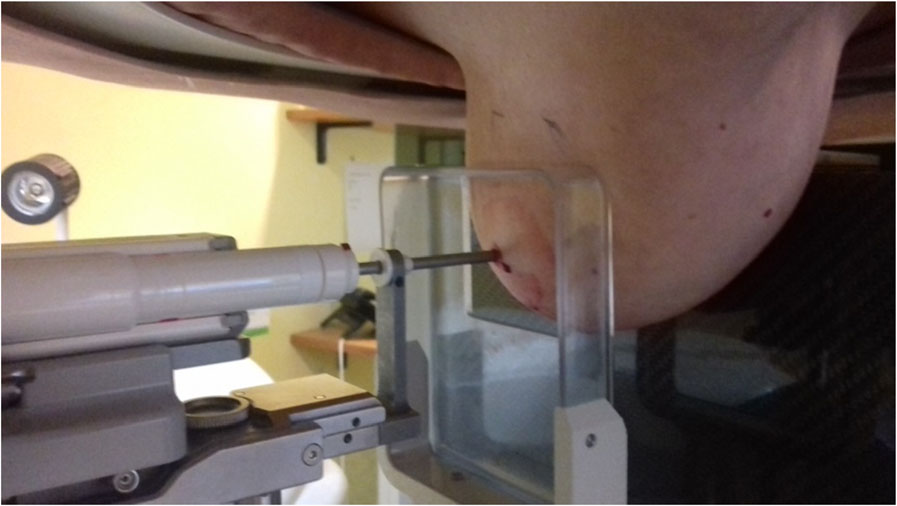
Mammography-guided (stereotactic) vacuum-assisted biopsy. The patient is in prone position lying on the table over the field of view of the image, with the breast pendent by gravity (she does not see the procedure). After local anaesthesia, the needle is guided to the lesion by the computer on the basis of specifically acquired mammographic images

**Fig. 3:**
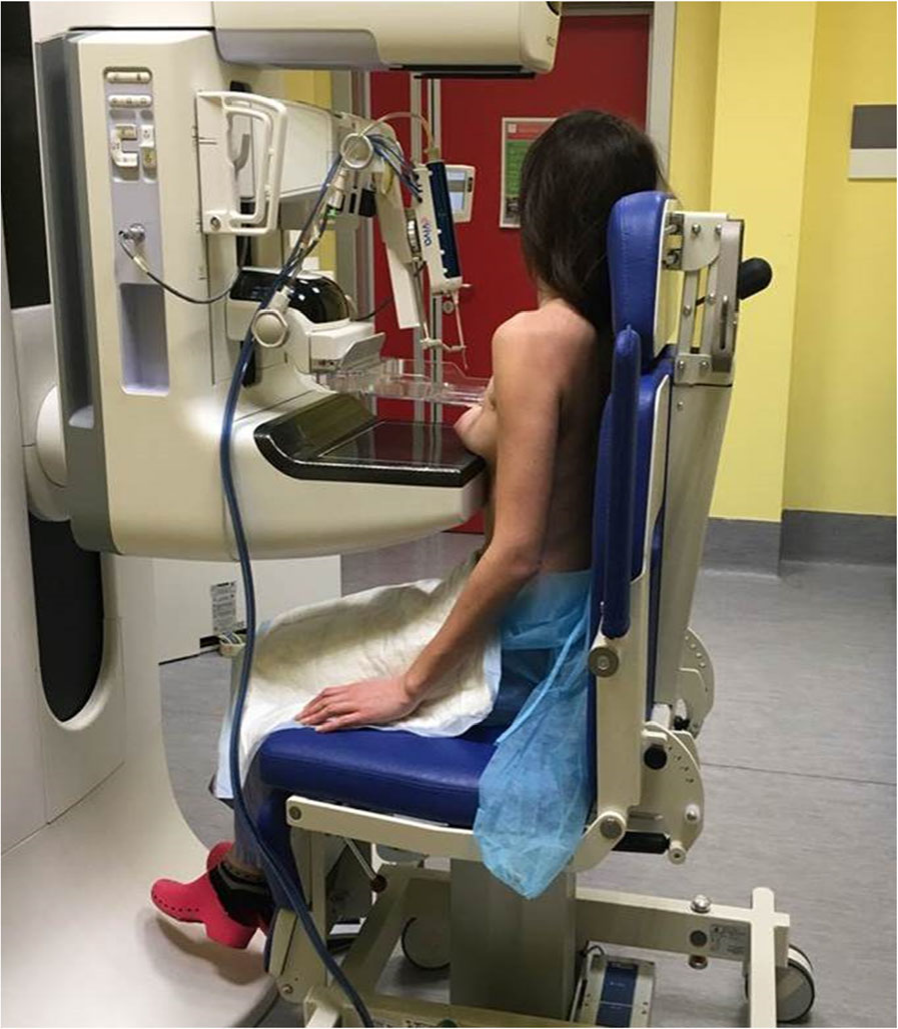
Mammography-guided (stereotactic) vacuum-assisted biopsy. The patient is sitting on a dedicated chair. After local anaesthesia, the needle is guided to the lesion by the computer on the basis of specifically acquired mammographic images

Mammographic guidance is the method of choice for lesions detected by mammography which do not have a clear correlate on US [51]. Most of these lesions are suspicious calcifications, architectural distortions, or small masses. The well-established method for mammography-guided biopsy is called *stereotaxis*, a term that refers to the need of two oblique projections providing a two-view (*stereo*) information to the operator [60, 61]. Stereotactic interventions are performed using dedicated prone tables, where the biopsy equipment is located below the patient, thus not visible to the patient during the procedure (Fig. 2), or upright mammographic add-on systems, with the patient usually sitting (Fig. 3) or lying in lateral decubitus during the procedure. A mild compression is required for breast immobilisation.

With dedicated prone systems, vasovagal reactions can be easily managed, but the upright add-on systems allow interventions to be performed in women who cannot lie prone due to orthopaedic problems. All stereotactic biopsies performed for calcifications should be followed by specimen radiography to document adequate sampling, *i.e.,*, the presence of calcifications within the samples. The duration of an uncomplicated stereotactic biopsy itself, excluding patient information before and after the procedure, histopathological correlation and reporting as well as preparation and cleaning of the room, is about 30 min.

In the last few years, mammography guidance can also be performed using digital tomosynthesis, a technique that provides images of thin slices of the breast [8, 62, 63]. These systems allow for precise localisation of the target with reduced time and radiation exposure compared to conventional stereotactic biopsy under mammographic guidance [64, 65]. The duration of a tomosynthesis-guided biopsy is about 10–15 min.

### MRI guidance

MRI-guided VAB is a safe and accurate procedure that is mandatory when suspicious lesions are visible on MRI only [48, 66–68]. Of note, among MRI-detected lesions, 46 to 71% may be revealed by a subsequent targeted US (the so-called *targeted US* or *second*-*look US*), even if breast US performed before MRI did not detect any abnormalities [69–71]. Importantly, identifying an MRI-detected lesion on US allows the biopsy to be performed under US guidance, in an easy, fast, comfortable, and cheap way [10]. Thus, the need for MRI-guided procedures is relatively limited: even large tertiary breast care institutions account for less than five patients per month [48]. It has been suggested that a high number of specimens may decrease underestimation rates of MRI-guided vacuum-assisted biopsy [72]. A large European multicentre study on MRI-guided biopsy of 538 lesions [73] reported a success rate of 96%, without any false negative among the 517 successful procedures at a median follow-up of 32 months (range 24–48 months).

Of course, any contraindications to MRI and/or gadolinium-based contrast administration must be checked as for any contrast-enhanced breast MRI [9], taking into consideration any interval change (*e.g.,* implantation of MRI-unsafe devices) between the previous breast MRI exam and the current MRI-guided procedure to be performed.

It is important to note that devices and software for MRI-guided interventions are not ubiquitously available across countries, and women cannot easily access these procedures partly due to diversity in public/insurance coverage and reimbursement policy. A EUSOBI survey among European radiologists [74] showed that only about one third of 177 responders practice MRI-guided interventions.

**Fig. 4:**
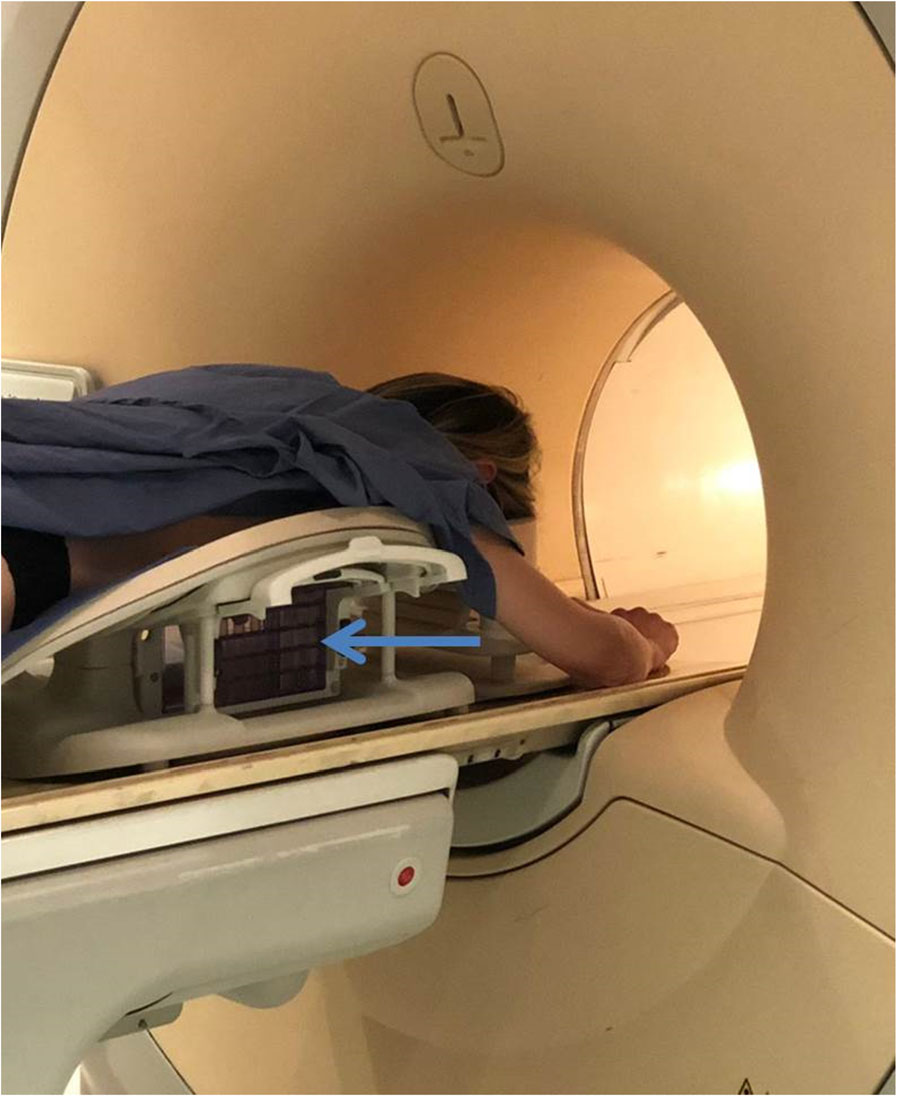
Magnetic resonance imaging-guided vacuum-assisted biopsy. The patient is prone, positioned on a dedicated coil that allows to insert the needle through a greed (light blue arrow), shown in the figure outside the magnet. After local anaesthesia, the needle is guided to the lesion on the basis of specifically acquired magnetic resonance images. To conclude the procedure, the patient has to enter and exit the magnet at least three times (see text)

MRI-guided biopsy is performed on the MRI table, with a sequence of movements inside and outside the magnet. The patient is placed in prone position with the targeted breast gently compressed in a dedicated biopsy grid (Fig. 4). A dedicated radiofrequency coil is required to enable image acquisition and to guide needle placement. The procedure is safe and accurate in specialised centres and relatively fast, but longer if compared to other imaging-guided procedures. Notably, there may be limited access for lesions close to the chest wall (depending on the biopsy coil setup) or close to the nipple as well as for lesions in small breasts. An MRI-guided biopsy is a multistep procedure and may exceed 30-min magnet time, considering the following steps:

1. The patient is placed inside the magnet.
2. Pre-contrast and immediate post-contrast T1-weighed series are acquired for lesion localisation.
3. The patient is moved out of the magnet to percutaneously access the lesion (from the side).
4. The patient is again moved inside the magnet for checking the biopsy device position.
5. The patient is moved out of the magnet to perform the biopsy and deploying the marker.
6. The patient is then finally moved inside the magnet to evaluate marker localisation and possible complications such as bleeding

Of note, additional time is needed for compressing the breast after biopsy (especially if bleeding occurs) and for cleaning, before the next patient can be examined.

***Note C.*** *If a suspicious lesion is visible on US, the best choice is to perform the biopsy under US guidance. If a suspicious lesion is visible only on mammography or tomosynthesis, these methods have to be used for guiding the biopsy. If a suspicious lesion is visible only on MRI, MRI-guided biopsy should be performed; if your centre has no possibility to perform an MRI-guided biopsy, ask your radiologist for a referral to a centre offering this procedure.*

## Axillary lymph node needle biopsy

Axillary lymph node assessment is an integral part of preoperative staging in patients with newly diagnosed invasive breast cancer [12]. Information about possible metastatic involvement of axillary lymph nodes can help avoid unnecessary procedures, triaging patients directly to axillary lymph node dissection [13]. Axillary lymph node US is the easiest way to identify abnormal lymph nodes. Tissue sampling with FNS or CNB is similar as in the breast. CNB seems to be more accurate than FNS in the diagnosis of axillary lymph node metastasis [14]. The incidence of adverse events is rather low; however, complications can be more severe than those of breast tissue sampling. After a biopsy, a clip may be placed in the biopsied lymph node, and marking of lymph nodes with clips and other devices is currently under investigation.

## Pathological-radiological correlation

For any combination of guidance methods and needle types, *concordance between the cytological or pathological result and the radiological appearance of the targeted lesion has to be verified*. *This mandatory task must be performed by the professional who performed the image*-*guided sampling*, *usually a breast radiologist*, *in collaboration with the reporting pathologist* [75–78].

In most cases, the radiologist will consider the cytology or pathology result as concordant with imaging findings, especially when CNB or VAB has been performed. However, in the case of inadequate sampling (more frequent in the case of FNS) or discordance between cytology/pathology and imaging findings, a repeat biopsy using the same or a different biopsy method can be considered, which is often after discussion in the multidisciplinary meeting. In the case of findings highly suspicious for malignancy on US, mammography/tomosynthesis, or MRI with discordant benign findings at FNS/CNB/VAB, it is also possible to go directly for surgical removal, after preoperative image-guided localisation, if the lesion is nonpalpable.

## Possible side effects and complications including neoplastic seeding

Percutaneous breast biopsies are minimally invasive and in general very safe, with severe complications requiring treatment being exceedingly rare [15, 34]. Common side effects include minor pain and bruising [41]; other complications are large haematomas or infections, at a rate of one every 1000 procedures [77, 79].

The risk of bleeding will increase with the needle diameter and the amount of sampled tissue [15]. Bleeding after biopsy is usually self-limited or limited by manual compression for 5–10 min (the time may be longer in the case of arterial bleeding) but may produce mild discomfort and pain for several days. *Severe bleeding requiring surgical intervention is very rare*. *Careful screening for bleeding disorders*, *avoidance of vessels when choosing the needle track* (*possible only under US guidance using colour*-*Doppler imaging*), *and adequate compression after the procedure reduce the probability of severe bleeding*.

*Pseudoaneurysm* (also called false aneurysm) of breast vessels is a very rare complication after either CNB or VAB, reported in the literature mainly as case reports. It is due to blood leaking from a breach in the arterial wall, contained by the adventitia or surrounding perivascular soft tissue. An enlarging mass a few days after the procedure, showing blood flow inside, is the commonest presentation. Management options include observation, US-guided focused compression, thrombin injection, open surgical repair, and percutaneous embolisation [80].

As with any percutaneous intervention, a low risk of *infection* does exist, so using sterile packed devices and keeping the environment clean as much as possible is important. The risk of infection after breast biopsies is extremely rare, reported to be 0.1% for US-guided biopsies [81]. They are mostly limited to skin or soft tissue and highly responsive to oral antibiotics. The risk of infection may be higher in patients with diabetes or a compromised immune system.

The risk of *mechanical displacement of malignant cells along the biopsy tract* can very rarely occur and is referred to as *neoplastic seeding*. The incidence of this event was reported to be 2 of 1644 biopsies [82] or, more recently, 8 of 4010 biopsies, [83], meaning that one or two of such events are expected every one thousand biopsies, likely because the displaced tumour cells are usually not viable [84]. Their biological significance is disputed, since such seeding is mostly located in the skin and detected early and thus removed without consequences. A retrospective analysis [85] including 719 patients after conserving surgery and radiotherapy for stage I and II breast cancer (189 patients having preoperative CNB and 530 without, with a median follow-up of 78 and 71 months, respectively) did not show increase of the local recurrence rate for the CNB group.

A study [86] reported an increase in *distant metastases* at long-term follow-up for a cohort of patients who underwent CNB versus FNS. However, the study has relevant limitations, the most important being the retrospective design, the small sample size and the times of cancer diagnosis of the two cohorts (203 patients diagnosed from 1991 to 1995 for CNB; 181 patients diagnosed from 1971 to 1976 for FNS). The authors adjusted for the difference in treatment over time. However, the 20-year time difference implies a greater sensitivity for metastases in the CNB cohort as a non-negligible source of bias [87]. In addition, no prospective studies replicated these findings.

A severe, but extremely rare complication of a free-hand or US-guided breast biopsy is the development of a chest wall injury, for example a *pneumothorax*, especially if an inexperienced examiner uses an improperly steep angle for access [88]. A pneumothorax is an abnormal collection of air between the lung and the chest wall and presents with sudden onset of sharp, one-sided chest pain and shortness of breath. The risk is practically null with mammography or MRI-guided biopsy, because the needle is introduced parallel to the chest wall.

***Note D.*** *Percutaneous breast biopsy is a safe procedure, with an extremely low risk of serious complications needing surgery or emergency assistance. Mild complications are also very rare: a certain degree of bleeding is the most common side-effect, usually self-limited; skin or soft-breast tissue infections respond well to oral antibiotics. The probability of neoplastic seeding along the biopsy tract is 2 of 1000 biopsies or lower and considered irrelevant due to the adjuvant radiation and pharmacological treatment in case of breast cancer. All mentioned complications are less frequent than complications under surgical biopsy with or without full anaesthesia.*

## Preparation for image-guided breast interventions

The planned procedure, including the rationale for performing the biopsy/localisation, potential benefits, possible complications, and likely outcomes must be explained in detail to the patient and informed consent should be obtained in advance (*e.g.,* when planning the procedure or 1−2 days before the procedure) or immediately before the procedure [51, 58].

### Bleeding disorders and antiplatelet and anticoagulation therapies

To minimise the risk of bleeding, patients scheduled for percutaneous breast biopsy should be *screened for bleeding disorders and antiplatelet and anticoagulation therapy*. For CNB, blood tests are considered necessary only in the case of a personal or family history of anticoagulation therapy. For VAB, most institutions prefer having blood testing performed, but in the absence of positive history, to perform the procedure without blood testing is considered acceptable. Breast biopsy is considered a percutaneous procedure with low risk of bleeding. If necessary, breast biopsies can safely be performed even in patients receiving anticoagulation treatment [41, 89]. Depending on national or local recommendations, anticoagulant agents like warfarin may be discontinued some days before and resumed 12 h after the procedure. For patients who cannot be off anticoagulation therapy, heparin bridge therapy may be planned by the clinical team. Low-dose or high-dose acetylsalicylic acid (*aspirin*) treatment does not need to be blocked while *clopidogrel* may be discontinued some days before and resumed immediately after the procedure [90]. Specific recommendations are in use for newer anticoagulant drugs. Of course, these recommendations are more important for VAB than for CNB; in general, the larger the needle and the higher the number of planned samples, the more attention should be paid to bleeding disorders and antiplatelet or anticoagulation therapies. All decisions to discontinue therapies should be taken in close collaboration with the prescribing physician.

### Pregnancy

In case of pregnancy, mammographic and MRI-guided procedures are possible but the indications should be adequately reviewed on a case-by-case basis.

### MRI-related contraindications

Patients planned for MRI-guided biopsy should undergo the usual precautions including screening for MRI-unsafe or MRI-conditional implants, prior reactions to contrast agent administration, and renal function impairment, as suggested by the EUSOBI recommendations for breast MRI [9].

The patients do not have to fast on the day of the procedure or discontinue other medications than those related to antiplatelet/anticoagulation therapy, as explained above.

***Note E.*** *Informed consent is required before a breast biopsy. Management of antiplatelet/anticoagulation therapy may be adopted. Women should be informed of possible adverse events and any allergies to local anaesthetic drugs should be verified. If an MRI-guided biopsy is planned, allergies to intravenous contrast agents as well as contraindications to MRI should be verified. Informed consent also includes communication between the radiologist and the patient (see the next paragraph).*

## Patient’s experience and communication between radiologist and patient

The patient should be informed about the reasons for performing a needle biopsy or a localisation procedure.

Before the procedure, *informed consent* must be obtained according to national/local regulations.^1^ Information must be given on the devices (needles) used for sampling and for guiding the sample, on the frequency of adverse events, side effects, and complications, better if using natural frequencies instead of percentages or complex epidemiological indices, including the need for repeat biopsy or surgical excision in cases of inadequate sampling, poor radiological-pathological concordance, or pathological diagnosis of high-risk/B3 lesions, as explained above.

Patients should be informed about the possible necessity of placing a marker clip in the biopsy site, taking into account that some women may have objections against potentially permanent placed foreign bodies in their breasts. In those cases, precise information on the risks associated with not using these markers has to be provided to the patient, especially the risk of missing a small lesion requiring surgery that may not be visible after the biopsy.

*Efforts should be made to reduce the patient*’*s anxiety prior to the procedure*, as the anticipated pain strongly correlates with the level of pain experienced by the patient during the procedure, so that communication with patients before biopsy regarding minimal average pain reported during biopsy is a good strategy [91]. The quality of human communication between the radiologist and the patient has been demonstrated to impact on patient’s anxiety [92]. A study reported that listening to guided meditation lowered biopsy pain during biopsy and that meditation and music reduced patient anxiety and fatigue without a negative impact on radiologist-patient communication [93]. We highlight that a good organisation of the medical team, a calm and efficient workflow, and a constant communication with the patient before, during, and after the procedure ensure a high compliance.

The experience of a patient needing a biopsy differs according to the image guidance adopted, as explained in the previous sections. While US-guided is in most cases a straightforward and fast procedure lasting no more than 15 min, mammography/tomosynthesis- and MRI-guided biopsies require preparatory technical measures aiming at the exact lesion localisation, leading to procedural times that can exceed 30 min and increase patient anxiety. Some evidence exists that tomosynthesis guidance can reduce the procedural time in comparison with stereotactic guidance [64]; one study [65] comparing 706 procedures under tomosynthesis guidance versus 439 procedures under stereotactic guidance showed an over 50% reduction in procedural time and about 75% reduction in radiation exposure.

Whichever combination of guidance and needle is used, once the patient is positioned and the best and safest needle access route to the lesion has been chosen, the corresponding skin is cleaned and disinfected. Whereas for FNS the use of local anaesthesia is optional (the size of the needle used is similar to an anaesthesia needle and the anaesthetic liquid could interfere with cytological aspiration), all other types of percutaneous breast biopsy are usually performed under local anaesthesia.

For superficial anaesthesia, lidocaine buffered in sodium bicarbonate may be used to reduce the initial stinging sensation of the lidocaine injection [41, 58]. In the case of allergy to lidocaine, alternative drugs have to be used. Even with optimal local anaesthesia, some discomfort or pain may be felt during needle insertion and tissue sampling, which will vary significantly from patient to patient [91].

At the completion of the biopsy, local manual compression, usually for 5 min, as well as cooling, may be applied to the biopsy site to achieve haemostasis and to minimise the amount of bleeding. In addition, the application of a compression bandage can be applied, in particular in the case of moderate or severe bleeding,

***Note F.*** *Patients’ experience will be different depending on the type of device used for sampling and the imaging guidance adopted. A percutaneous biopsy may last from 15 to 45 min and is generally well tolerated. The position will be the same as the diagnostic examination for US (supine) or MRI-guided biopsy (prone) and may be prone, seated, or in lateral decubitus for mammography/tomosynthesis-guided biopsy, according to the adopted system. Communication between the radiologist and the patient is crucial for anxiety reduction.*

## Post-procedural recommendations and communication of results

Following the biopsy procedure and after achieving haemostasis with manual compression, wound cleaning, and bandage, the patient is observed for 15–60 min in the radiology suite, depending on the type of procedure and the patient’s profile. Subsequently, she can be discharged with appropriate instructions, including possible pain and bleeding and corresponding remedies, ban of water immersion (*e.g.,* swimming) and strenuous exercise for at least 3–5 days following the biopsy.

Whenever possible, biopsy results as well as recommendations for further management (*e.g.,* treatment or follow-up) should be discussed with the patient in person, usually during the visit after the biopsy. Timing of this visit must strike a balance between minimising the waiting time for the patient and its associated anxiety and the necessity to allow for enough time to have pathology results including, if necessary, additional immune-histological stains. In the cases requiring treatment, the visit after the interventional procedure should include the results from the multidisciplinary conference, at which concordance of imaging and histological findings is assessed and subsequent management is established.

Some centres have developed rapid diagnosis as “*one*-*stop breast clinics*”, providing the results of the biopsy immediately after the procedure. This type of management has been proven to be cost-effective and accurate [94]. However, efforts should be made to decrease distress and anxiety of the patients also in this setting in regards to the quality of the patient-doctor relationship and doctors’ interpersonal skills, doctors’ availability, and waiting time [95], which are important general aspects to be improved in breast image-guided interventions.

***Note G.*** *At the end of biopsy, the patient is observed in the radiology department for 15–60 min. She can then be discharged and adequate instructions on common side effects and complications, as well as corresponding remedies are suggested. The next visit is planned for communication and discussion of the results, including the radiologist who performed the procedure and participated in the assessment of concordance between cytology/pathology diagnosis and lesion appearance at breast imaging.*

## Preoperative image-guided localisation of nonpalpable breast lesions

Nonpalpable, clinically occult breast lesions amenable to surgery need image-guided preoperative localisation. This allows guidance of surgeons to the lesion for a safe and effective intervention aiming at obtaining both a complete removal (clear margins) and a good cosmetic result [2, 3, 96]. Radiologists play a crucial role in this.

Different localisation methods currently exist and are variably used in different institutions depending on personal choices, skills, and available technologies. The image guidance adopted should be the easiest method whereby the lesion (or the marker left after the biopsy) can be identified with certainty. In case of findings only visible on mammography/tomosynthesis (*e.g.,* calcifications) or lesions revealed only on MRI, mammography/tomosynthesis or MRI guidance have to be used, respectively. This is why post-interventional marker placement is encouraged: the marker allows a presurgical localisation by US-guided marker identification. The position of the patient is the same in which the biopsy was performed; supine for US-guided methods; upright, prone, or in lateral decubitus for mammography/tomosynthesis-guided methods; and prone for MRI-guided methods.

Methods for preoperative localisation of nonpalpable breast lesions are *carbon marking*, *wire localisation*, *radiotracer injection* (usually called *radio*-*guided occult lesion localisation*, ROLL), *radioactive seed localisation*, and *magnetic seed localisation* (Table 2).

**Table 2:** Current options for image-guided localisation of nonpalpable breast lesions

Method	Notes
Carbon marking	Old method; however, needing specific local experience. Surgery up to 1 month after. If not removed, it can mimic malignancy.
Wire localisation	Mostly used. Surgery at the same day or the day after. Possible vasovagal reactions, wire rupture or migration.
Radio-guided localisation	Specific local experience is required. Surgery within 24 h after. Radiation exposure (low-dose). Higher cost than wire localisation.
Radioactive seed localisation	Specific local experience is required. Interval time from procedure to surgery possibly longer than with radio-guided localisation. Radiation exposure (low-dose). Higher cost than wire localisation.
Magnetic seed localisation	Recently introduced. Specific local experience is required. Surgery up to 1 month after. Higher cost than radio-guided and radioactive seed localisation. Magnetic seeds to be completely removed to avoid artefacts in breast magnetic resonance imaging examinations.

### Carbon marking

Carbon marking is a long-standing method consisting of injection of sterile charcoal powder diluted with saline solution near to a nonpalpable breast lesion under US or mammographic guidance, depending on how the biopsy was performed [97, 98]. A dark trail is created from the lesion to the skin leaving a visible spot. The surgeon is guided by the presence of the carbon suspension, which should be removed during the operation. Surgery may be planned up to 1 month from the carbon injection. Needle obstruction by the charcoal powder may occur but it can be remedied. The probability of failure (*i.e.,* of not surgically removing the lesion) is about 1 in every 100 procedures [97]. In the cases which do not proceed to surgical removal of the carbon (*e.g.,* when a benign diagnosis is obtained with the biopsy), foreign-body giant-cell reactions that may mimic malignancy on mammography and US may occur (about 3 cases for every 100 carbon localisations which are not surgically excised) [99]. The method is still used in some centres but less adopted than wire localisation.

### Wire localisation

It is the commonest method worldwide. A 3–15 cm wire, usually with a terminal hook or pigtail, is inserted through the skin by the radiologist using a co-axial needle introducer and anchored to the lesion or nearby through a 16–21-gauge needle that works as introducer. A variable part of the wire comes out of the skin and is taped or covered until the patient is transferred to the operating room and the surgeon removes it together with a variable amount of breast tissue. Wire localisation is a safe, cost-effective, and standardised technique which may be performed under US, mammography/tomosynthesis, or MRI guidance. It strongly reduces the even low risk of incorrect localisation associated with use of marking fluids (carbon suspension or radioactive tracers) that might distribute along the septa, resulting into imprecision. The main complications are patient discomfort such as vasovagal reactions (from mild light-headedness to syncope, the latter only in 1% of cases), reported in up to 7–10% of patients and less frequent for US than for mammography guidance), wire rupture or migration [2, 3, 100–104]. The guidewire should be positioned ideally at the day of surgery or the day before surgery, an issue which must be considered for surgical scheduling. This timing is important to avoid possible infections and wire migration.

### Radio-guided occult lesion localisation

ROLL consists in the injection of 0.2–0.3 mL of human serum albumin labelled with radioactive technetium (^99m^Tc) inside the tumour [105]. US, mammography, or MRI guidance can be used. The radiation dose is that due to a radioactivity of about 7–10 MBq, a dose equivalent to 1–2% of that used for a whole-body bone scintigraphy (600 MBq) [106]. A scintigraphy scan of the breast is then obtained to check the correct inoculation of the tracer by comparison between its position and the localisation of the lesion on mammograms and/or US. During the surgery, performed no later than 24 h after the injection, the tumour is detected by a gamma probe, used by the surgeon to locate the lesion, guide the removal, and verify the removed specimen and the surgical bed. Experience is needed because the tracer can be dispersed in the ducts and identification of the lesion may fail. Studies showed a correct positioning of the radiotracer in 94.6–99.6% of cases [107, 108], allowing for tumour excision with negative margins in 92% of cases [108]. A meta-analysis of four randomised controlled trials totalling 449 patients [109] showed that accurate localisation, peri-procedural complications, volume and weight of the excised occult breast lesion, and reoperation rate after wire localisation and ROLL were similar; duration of localisation and surgical excision was shorter for ROLL. Of note, an additional radiotracer (carried by micromolecules instead of macromolecules used for ROLL) can be injected near to the tumour to be drained in the sentinel node, that will be identified by the gamma probe and then biopsied during the surgical intervention removing the primary tumour (*sentinel node and occult lesion localisation*, SNOLL) [108]. However, performances of both procedures may be dependent on local experience and their cost is higher than that of wire localisation.

### Radioactive seed localisation

For this approach, radioactive seeds are positioned inside the tumour under US, mammography/tomosynthesis, or MRI guidance. These seeds are small titanium radioactive ^125^I parts (about 0.5 to 1 × 4 to 5 mm), commonly used for brachytherapy (a modality of radiation therapy that brings many of these seeds inside the tumour) [110]. One seed has a radioactivity of about 20–30 MBq, a dose equivalent to 3–5% of that used for a whole-body bone scintigraphy. A meta-analysis of six studies totalling 1611 patients [111] showed an overall complete resection rate ranging from 73 to 97% (the inferior rate was 90% for studies including over 300 patients); the risk of seed migration was lower than 1%, that of failure of seed placement from 0 to 7%. The authors concluded that radioactive seed localisation of nonpalpable breast lesions is safe and accurate, with an efficacy similar to that of ROLL and the advantages of possible longer time between localisation and surgery allowed, without risk of dispersion through the ductal tree [112]. A potential for reduction of excised volume in the case of DCIS has been recently showed using multiple seeds [113]. Obviously, for ROLL and radioactive seed localisation, a strict cooperation between the radiology and nuclear medicine/radiotherapy departments and the availability of a gamma probe in the operating room are necessary. According to local regulations, especially in the case of planned travel of the patient receiving the seed, an official signed document containing information on the date of the procedure and the associated radioactivity level can be given to the patient.

### Magnetic seed localisation

This recent localisation method uses 5 × 1 mm paramagnetic steel and iron oxide cylindrical seed, readily visible on mammography/tomosynthesis and US, supplied preloaded into an 18-gauge 20-cm needle. The seed is detectable using a dedicated magnetic probe. The first published reports [114–116] indicated its potential to be a good alternative to the other methods being radiation free, and not requiring a short time between localisation and surgery (up to 30 days interval). The cost is certainly higher than that of wire or radioactive localisation techniques. Future large studies are needed to confirm its use in common clinical practice. Of note, magnetic seeds need to be completely removed to avoid artefacts in breast MRI examinations.

We emphasise that *the current recommended and most commonly used method is wire localisation*. Whether alternative approaches may lead to equivalent or even better results has not been established yet and depends on local experience. In addition, methods using radioactive tracers or seeds have the drawback of radiation exposure even though low doses are used. A meta-analysis from the Cochrane Database published in 2015 [117], considering eight randomised controlled trials investigating wire localisation, ROLL, and radioactive seed localisation concluded that *there is no clear evidence to support one guided technique over another*. The authors *support the continued use of wire localisation as a safe and tested technique that allows for flexibility in selected cases when faced with extensive microcalcifications*.

Whichever localisation technique is used, additional marking of the skin directly over the nonpalpable breast lesion and measurement of the depth of the lesion in the supine operative position using US (when the lesion is US-detectable) is of great help to the surgeon. Communication between the radiologist and the surgeon is also crucial for choosing the best method for localisation, considering the surgical approach to be used.

***Note H.*** *When the pathological result of a biopsied nonpalpable lesion requires surgical excision, a preoperative image-guided localisation is performed by the radiologist. This will allow the surgeon to access the lesion accurately and to remove it. Percutaneous localisation can be performed under US, mammography/tomosynthesis, and MRI guidance, whichever is more suitable and cost-effective for the specific case.*

## Conclusions

Image-guided percutaneous needle biopsy represents a fundamental technique to characterise the nature of suspicious breast abnormalities and has almost completely replaced the surgical biopsy for both palpable and nonpalpable lesions. Radiologists play a crucial role in the detection as well as diagnosis and management of breast disease. Depending on the patient profile and lesion characteristics, the radiologist will choose the best available biopsy system from FNS, CNB, or VAB. The choice will also depend on local experience and availability. When a lesion is visible on US, this is the preferred method for biopsy being most accessible, comfortable, and straightforward compared to other techniques. Minor pain and bleeding are possible post-biopsy sequelae, but the risk of severe complications is very low. Radiologic-pathologic correlation is essential for an accurate and successful conclusion of the diagnostic procedure.

Preoperative localisation of nonpalpable lesions amenable to surgery is available through US, mammography/tomosynthesis, or MRI guidance, typically by wire insertion.

## Frequently asked questions (FAQs)

1. *What is the difference between needle biopsy and excisional biopsy*?

Needle biopsies are percutaneous interventional procedures performed with a needle under the guidance of a breast imaging modality; depending on the needle size, local anaesthesia is administered. Excisional biopsy is a surgical operation aiming at removing the entire lesion to be analysed by the pathologist; it may be performed under local or general anaesthesia.

2. *What is the difference between FNS*, *CNB and VAB*?

FNS is percutaneous lesion sampling, usually performed under US guidance, using the same fine needles suitable for intramuscular injections or thinner needles, according to local experience. Once the needle is in the lesion, multiple cells are collected; the biological material is prepared as smears on glass slides and examined by the pathologist for a cytological diagnosis. CNB is a percutaneous procedure that uses a spring-loaded needle for “true cutting” tissue “cores” providing a histological tissue diagnosis. VAB performs a task similar to that of CNB using larger needles, facilitated by a vacuum-generating device that attracts the tissue towards the needle, allowing removal of larger amounts of tissue in comparison to CNB.

3. *Is breast needle biopsy a painful procedure*?

Minor pain and bruising can occur immediately at the end of the procedure and up to one or two days. If necessary, adequate analgesic support can be taken.

4. *Is local anaesthesia used during a breast biopsy*?

Yes, local anaesthesia is always administered before core needle biopsy (CNB) or vacuum-assisted biopsy (VAB). Before FNS, as the procedure for delivering anaesthesia is not different from collecting cells, local anaesthesia is optional.

5. *Is there any risk of dissemination or activation of cancer cells with image*-*guided interventional procedures*?

The risk of mechanical displacement of malignant cells along the biopsy tract can rarely occur and is referred to as “neoplastic seeding”. It has been reported to happen in less than 2 cases every 1000 biopsies. It is considered oncologically irrelevant due the high probability of lack of viability of the displaced cells and to the possible addition of adjuvant radiation and pharmacological treatment in the case of breast cancer. In fact, preoperative needle biopsy does not increase local recurrence rate in breast cancer patients. In addition, if seeding does occur, it is mostly seen in the skin, which is detected early and easily treated, with low clinical importance.

6. *Can needle biopsies provoke infections*?

A very low risk of infection accounting for 1 case every 1000 procedures does exist, and adherence to sterile working conditions is important. Infectious complications are limited to skin or soft tissue and respond well to oral antibiotics. The risk of infection may be higher in patients with diabetes or compromised immune systems.

7. *How much waiting time to get the result of the needle biopsy*?

In some centres, the result can be available very soon after the procedure. Otherwise, the cytology/pathology report should be available within 1 or 2 weeks after the procedure. However, special cases (*e.g.,* when a pathological-radiological discordance happens, requiring consultation among the specialists and potential re-reading by the pathologist) may need longer time to provide results.

## Data Availability

Not applicable

## Abbreviations

CNB: Core needle biopsy
DCIS: Ductal carcinoma in situ
FNS: Fine-needle sampling
MRI: Magnetic resonance imaging
ROLL: Radio-guided occult lesion localisation
US: Ultrasound
VAB: Vacuum-assisted biopsy

## Footnotes

1 Rules for the informed consent required for needle tissue sampling and lesion localisation can be different according to national, regional, or local regulations, including written informed consent after presentation of written information about advantages and disadvantages associated to the procedure or oral informed consent described in the radiological report.
